# Exploring the impact of integrated polyvagal exercises and knee reinforcement in females with grade II knee osteoarthritis: a randomized controlled trial

**DOI:** 10.1038/s41598-023-45908-4

**Published:** 2023-11-03

**Authors:** Moattar Raza Rizvi, Ankita Sharma, Shahnaz Hasan, Fuzail Ahmad, Mohammad Rehan Asad, Amir Iqbal, Ahmad H. Alghadir

**Affiliations:** 1 Department of Physiotherapy, School of Allied Health Science, Manav Rachna International Institute of Research and Studies, Faridabad, 121004 India; 2 Department of Physical Therapy and Health Rehabilitation, College of Applied Medical Sciences, Majmaah University, Al-Majmaah, 11952 Saudi Arabia; 3 Respiratory Care Department, College of Applied Sciences, Almaarefa University, Ad Diriyah, 13713 Saudi Arabia; 4 Department of Basic Sciences, College of Medicine, Majmaah University, Al-Majmaah, 15341 Saudi Arabia; 5 Department of Rehabilitation Sciences, College of Applied Medical Sciences, King Saud University, P.O. Box. 10219, Riyadh, 11433 Saudi Arabia

**Keywords:** Musculoskeletal system, Osteoarthritis, Health care, Rheumatology

## Abstract

This study aimed to compare the effects of knee strengthening exercises to those of polyvagal theory–based exercises combined with knee strengthening exercises on selected outcomes in women with grade II knee osteoarthritis (OA). A randomized controlled trial was conducted, in which 60 female participants diagnosed with grade II knee OA, with a mean age of 57.27 ± 7.81 years and knee pain rated between 4 and 7 on the visual analog scale (VAS), were assigned to either the knee strengthening exercise group (Group 1, n = 30) or the polyvagal theory–based exercise plus knee strengthening exercise group (Group 2, n = 30). Pre- and posttreatment assessment of outcome variables, including WOMAC scores (joint pain, joint stiffness, functional limitations, and the overall index), WHOQOL scores (overall quality of life, general health, physical, psychological, social, and environmental domains), and heart rate variability (HRV, time and frequency domains), were analyzed. Group 2 demonstrated significantly greater reductions in joint pain, stiffness, and functional limitations than Group 1 after the intervention. Group 2 presented with significantly improved WOMAC scores, indicating better overall outcomes. Group 2 showed significant improvements in the psychological and social domains regarding quality of life. There were no significant differences in the physical domain or the environmental domain. Group 2 showed a significant increase in high-frequency power (HF) and a significant decrease in the LF/HF ratio, suggesting improved autonomic regulation. A combination of polyvagal exercise and knee strengthening training resulted in superior outcomes compared to knee strengthening exercises alone in women with grade II knee OA. These findings support the potential effectiveness of incorporating polyvagal exercises as an adjunctive intervention for osteoarthritis management.

## Introduction

Osteoarthritis (OA) is one of the most prevalent degenerative conditions resulting in disability, particularly among the elderly population. OA is the most common articular disease in the developed world and a leading cause of chronic disability, primarily due to knee OA and hip OA^1^. The prevalence of osteoarthritis (OA) is significantly higher in women than in men, and the disease significantly affects both physical and autonomic functions^2^.

Women are particularly susceptible to OA, accounting for approximately 60% of all OA patients^3^. Age is a major risk factor, with the prevalence in women escalating from approximately 10% at ages 25–34 to over 50% at ages 75 and older^2^. Exercise plays a pivotal role in sustaining overall well-being and enhancing quality of life, particularly among older women^4^. The physical implications of OA in women include pain, stiffness, limited mobility, and a reduced capacity to perform daily activities, leading to compromised quality of life^5^. Autonomic dysfunction, characterized by irregularities in heart rate variability and blood pressure, has been reported in patients with OA, suggesting a link between chronic pain and cardiovascular risk ^6^.

The pathogenesis of knee OA involves a complex interplay of mechanical, genetic, and biochemical factors. It typically begins with cartilage degradation, leading to changes in the subchondral bone, inflammation, and alterations in joint tissues. As the cartilage wears down, the joint space narrows, bone spurs (osteophytes) may form, and the synovium (joint lining) may become inflamed. These processes collectively contribute to pain, stiffness, and functional limitations associated with knee OA^7^. The inflammatory response can be sustained by the release of additional pro-inflammatory factors from immune cells that have infiltrated the site, specifically neutrophils and monocytes. The persistent inflammatory response is a factor contributing to the degradation of cartilage and other joint components, exacerbating the discomfort and functional limitations associated with knee OA^8^. Grade II OA is characterized by mild to moderate joint damage. At this stage, the joint deterioration and functional limitations are significant enough to warrant intervention, yet the condition has not progressed to the extent that more invasive treatments, such as surgery, become necessary. Strengthening exercises can help improve muscle strength and joint stability, while vagal nerve stimulation may aid in regulating the autonomic nervous system and reducing pain perception^9^.

Strength training exercises effectively manage knee OA by improving muscle strength and function, thereby reducing pain and enhancing quality of life^10^. Studies consistently reveal increased rates of depression among individuals with OA compared to the general population. The chronic pain, functional limitations, and reduced quality of life associated with OA can contribute to the development or exacerbation of depressive symptoms^11^. According to the polyvagal theory, the vagus nerve plays a role in a hierarchical model of reactions to stress and social interaction, which consists of two primary branches: the ventral vagus and the dorsal vagus. Vagal nerve stimulation (VNS) is employed to regulate the vagus nerve's function, aiming to achieve equilibrium between the ventral and dorsal vagus responses^9^. VNS has demonstrated the capacity to modulate neural activity, influence neurotransmitter release, and facilitate neuroplasticity. The research that has been conducted to date on VNS not only offers valuable insights into the complex interplay involving the vagus nerve, autonomic regulation, and social behavior but also sheds light on the intricate connection between VNS and the activity of norepinephrine, serotonin, and other neurotransmitters implicated in mood disorders and depression, which are common in patients with knee OA^12^.

Strengthening exercises and vagal nerve stimulation are noninvasive interventions that can be implemented without the need for medications or surgical procedures. This makes them attractive treatment options for individuals with grade II knee OA who prefer a conservative approach or have contraindications for other treatments. Combining strengthening exercises with polyvagal exercises may offer potential synergistic effects in the management of OA symptoms. Strengthening exercises can help improve joint stability, reduce stress on the joint, and enhance overall physical function. Vagal nerve stimulation techniques following polyvagal exercises may help regulate autonomic function, reduce pain perception, and promote relaxation and emotional well-being. This study hypothesized that the combination of these interventions would lead to improved overall outcomes for individuals diagnosed with grade II knee OA.

Based on the above, the primary objective of this study was to investigate the potential benefits of combining strengthening exercises with polyvagal theory–based exercises as a noninvasive intervention for females diagnosed with grade II knee OA. Specifically, we aimed to assess whether the synergistic effects of these interventions could lead to improved joint stability, reduced pain perception, enhanced autonomic regulation, and improved overall physical function and emotional well-being among females with Grade II knee OA.

## Materials and methods

### Study design

The study employed a two-arm parallel-group randomized comparative design. In this study, females (n = 60) diagnosed with grade II knee OA according to the Kellgren-Lawrence (KL) scale^13^ were randomized into two groups: Group 1 was assigned to perform knee strengthening exercises (n = 30), and Group 2 was assigned to perform both knee strengthening exercises and exercises based on polyvagal theory to stimulate the vagal nerve (n = 30).

### Sample size

G*Power software version 3.1.9.4 (Heinrich-Heine-Universität Düsseldorf, Germany) was used to calculate the sample size. An a priori power analysis was performed for a repeated-measures analysis of variance examining within-groups and between-groups interactions with two groups and two repeated measures. For an effect size = 0.25, α = 0.05 and power = 0.95, the required sample size was n = 54 in total or 27 participants in each group. Considering an anticipated dropout rate of 10%, our study was designed to include a total of n = 60 participants at the start, distributed evenly (n = 30 individuals in each group).

### Procedure

**Figure 1:**
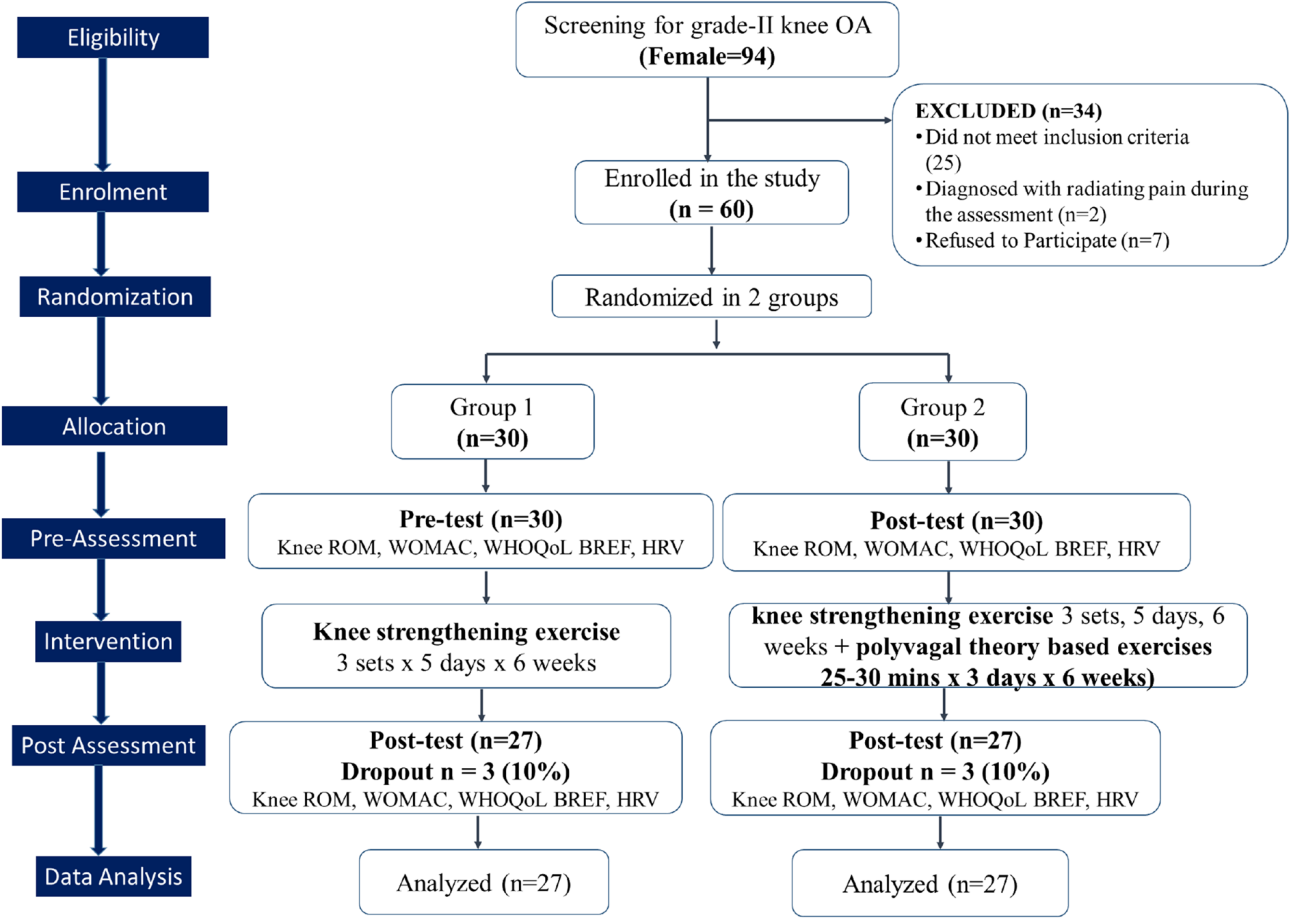
A CONSORT (2010) flow chart of the study design.

Ninety-four patients with complaints of knee pain underwent screening for grade II knee OA. However, thirty-four patients were excluded during the initial evaluation because they did not meet the inclusion criteria. The remaining 60 patients were randomly allocated into two intervention groups (with each group consisting of 30 patients): Group 1 performed only knee strengthening exercises, and Group 2 performed both knee strengthening exercises and polyvagal theory–based exercises. Randomization was performed at a 1:1 allocation ratio using sealed envelopes. Figure 1 illustrates the study procedures comprehensively through a CONSORT (2010) flow chart, outlining key stages such as assessment, enrollment, randomization, intervention allocation, follow-up, and data analysis.

### Study participants

#### Inclusion criteria

This study included women between 45 and 60 years of age who had knee pain and were diagnosed with grade II unilateral knee OA. The decision was made to exclusively enroll female patients in this study because the incidence of knee osteoarthritis is higher in women than in men. Additionally, this approach was taken to address the potential impact of sex-related differences in critical factors such as muscle strength and mass.

#### Exclusion criteria

The criteria for exclusion were as follows: (i) engagement in physical exercise training within the previous year; (ii) the presence of cardiovascular diseases and/or musculoskeletal disorders that would hinder participation in exercises; (iii) a radiographic grade of 1 or > 2 according to the Kellgren-Lawrence scale; (iv) a history of knee joint surgery; (v) consumption of nonsteroidal anti-inflammatory drugs within the preceding three months for knee pain; and (vi) intra-articular administration of hyaluronic acid and/or corticosteroids within the preceding six months.

### Outcome measures

#### Range of motion

The angle of knee flexion was measured using an app-based goniometer (DrGoniometers, CDM S.r.L), a tool that has been established as reliable and validated^14^. The participants lay in a supine position with the hip and knee of the affected limb flexed to a 90-degree angle while the feet were relaxed. The smartphone containing the accelerometer-based goniometer application was placed on the middle third of the anterior tibia and held in place by the examiner during the movement from the initial position to the final position. The "initial position" refers to the starting point where the hip and knee of the affected limb are flexed to a 90-degree angle, while the feet remain relaxed. A touch on the screen at this position established the 0-degree reference point. Subsequently, as the leg is moved in flexion, the examiner touches the screen again, representing the "terminal position." This terminal position signifies the final point of knee flexion in the movement and is measured while the hip maintains the same 90-degree angle of flexion.

#### Western Ontario and McMaster Universities Osteoarthritis Index (WOMAC)

The WOMAC is a self-administered validated questionnaire used for the assessment of hip and knee OA^15^. The WOMAC consists of 24 items divided into three subscales: pain (5 questions), stiffness (2 questions), and physical function (17 questions). The subscale scores can vary from 0 to 20 for pain, 0– to 8 for stiffness, and 0–68 for physical function, with higher scores (out of a maximum score of 96) indicating worse outcomes^16^. After signing the consent form, the patients filled out the questionnaire questions related to pain, function and stiffness at 0 days and after 6 weeks.

#### Abbreviated WHO quality of life scale (WHOQOL-Bref)

To assess patients’ quality of life, the WHOQOL-Bref questionnaire was used^17^. This contains questions focusing on QoL related to the physical, psychological, social and environmental domains. The questionnaire comprises 26 questions on the individual's self-perceived health and well-being over the previous two weeks. Responses to questions are on a 1–5 Likert scale, where 1 represents “disagree” or “not at all” and 5 represents “completely agree” or “extremely”. The possible score in each case ranges from 0 to 100 points, with 100 points indicating no restrictions at all due to the affected knee, while a score of 0 points indicates the worst possible health status.

#### Resting heart rate

Resting heart rate (RHR) was assessed using the Omron HEM 742 automated oscillometric sphygmomanometer^18^. Participants followed specific instructions, including resting quietly for at least five minutes in a calm environment with an empty bladder. They were also advised not to engage in any physical activity; smoke; or consume alcohol, coffee, or tea for specific timeframes before the measurement. During RHR measurement, participants sat with their backs supported and arms at a 90° angle. RHR was measured twice, 15 min apart, and we used the average of these two measurements in beats per minute (bpm).

#### Heart rate variability

On the day of the assessment, participants were asked to lie supine for 10 min or more to attain complete relaxation. Their skin was prepared by using a razor to remove the hair from the intended site of the HR sensor (in males only), cleaning the skin and drying it with gauze. They were asked to sit in a chair with armrests. The participants were instructed to wear an HR sensor on an elastic chest strap around the thorax in direct contact with the skin. RR intervals (the time elapsed between two successive R-waves of the QRS signal on the electrocardiogram) were recorded at a sampling frequency of 1000 Hz using the HR sensor (Polar H7, Polar Electro Oy, Kempele, Finland), which was connected via Bluetooth to an Android smartphone application (Elite HRV, Asheville, NC, USA). A digital electrocardiogram (ECG) was recorded for 5 min, and blood pressure was assessed in a sitting position^19^.

Time-domain analysis of HRV offers insights into the heart rate's temporal characteristics and the autonomic nervous system's influence on cardiac regulation. The time-domain measures of HRV were derived from a series of RR intervals. Various time-domain HRV indices were examined, including the mean of the normal-to-normal RR interval (MeanRR), the root mean square of successive differences between adjacent RR intervals (RMSSD), and the standard deviation of normal-to-normal RR intervals (SDNN). Additionally, frequency-domain indices were explored to analyze the distribution of power spectra across different frequency bands, revealing sympathetic-parasympathetic balance and autonomic heart rate control. These indices included total power (TP), reflecting overall signal variability (0.003–0.4 Hz); low-frequency spectral power (LF), associated with both sympathetic and parasympathetic activity (0.04 and 0.15 Hz); high-frequency spectral power (HF), mainly linked to parasympathetic activity (0.15 and 0.4 Hz); normalized unit of low frequency (nuLF), representing the percentage of total power in the LF band and indicating the sympathetic-parasympathetic balance; normalized unit of high frequency (nuHF), expressing the percentage of total power in the HF band and signifying parasympathetic activity; and the ratio of low frequency to high frequency (LF/HF), frequently employed as an indicator of sympathetic-parasympathetic balance. A higher LF/HF ratio implies greater sympathetic activity, while a lower ratio indicates higher parasympathetic activity. Collectively, these HRV variables provide comprehensive insights into the relative contributions of the sympathetic and parasympathetic branches of the autonomic nervous system to heart regulation. All HR recordings underwent meticulous visual inspection to ensure stationarity and were corrected for artifacts and ectopic beats using Kubios' built-in piecewise cubic spline interpolation^19^.

### Interventions

#### Knee strengthening exercise program

The targeted muscles, levels, types of exercises, and progression strategies are presented in Table 1. Each exercise was performed in 3 sets, and each repetition was held for 3 s. Both groups performed the exercises under the active supervision of the therapist 5 days a week for 6 weeks. The exercises were discontinued if an increase in pain was reported. The knee extensors, knee flexors, and gluteal muscles were targeted to improve the symptoms related to knee OA^20^.

**Table 1:** Knee strengthening exercise program for patients with KOA.

Targeted muscles	Level	Type of exercise	Sets	Reps	Progression
Knee extensors-open kinetic chain	Level 1	Isometric quads sets	3	10	Progress to level 2 when 3 sets of 10 is no longer challenging, and the patient is performing the activity with ease and good form
	Level 2	Terminal knee extension	3	10	Progress to level 3 when 3 sets of 10 are no longer challenging, and the patient is performing the activity with ease and good form
	Level 3	Terminal knee extension with weight	3	10	The weight was increased to 2 kgs depending upon the ease of the patient
Knee extensors-closed kinetic chain	Level 1	Terminal knee extension in standing with resistance band	3	10	The level of the resistance band is progressed sequentially progressing to squats
	Level 2	Supported half Squats	1	10	Deep squats with reduced weight
	Level 3	Step-ups with the affected limb	1	10	Progress to 13 cm step if knee pain was not increased
Hip extensors strengthening	Level 1	Supine glut sets	3	10	Progress to level 2 when 3 sets of 10 is no longer challenging, and the patient is performing the activity with ease and good form
	Level 2	Supine bridging	3	10	Progress to level 3 when 3 sets of 10 are no longer challenging, and the patient is performing an activity with ease and good form
	Level 3	Supine unilateral bridging	3	10	A 1 kg weight cuff was added when the patient was doing it with ease
Knee flexors strengthening	Level 1	Prone hamstrings curl up to 90 degrees	3	10	Progress to level 2 when 3 sets of 10 are no longer challenging, and the patient is performing the activity with ease and good form
	Level 2	Prone hamstrings curl with a weight	3	10	Progress to level 3 when 3 sets of 10 are no longer challenging, and the patient is performing the activity with ease and good form
	Level 3	Standing hip extension with resistance band	3	10	The resistance of the band was increased when the patient was able to perform the exercises well

#### Polyvagal exercise protocol

The polyvagal exercises were aimed at activating and regulating the autonomic nervous system, which plays a crucial role in physiological and emotional responses. These exercises were tailored and customized to stimulate the vagus nerve and thereby promote relaxation and improve social interaction following the principles of polyvagal theory^9,21^. The domains of exercise, types of exercise, procedure and duration of polyvagal exercise are presented in Table 2.

**Table 2:** Tailored polyvagal exercise protocol ^9,21^.

Domains of exercises	Type of exercise	Procedure	Duration
Sensory awareness exercises	Breathing exercises	In a calm position, focusing on breathing and keeping the awareness of inhalation and exhalation	20–30 breaths (2–3 min)
	Stand upright postures	Awareness of different standing postures like standing tall, standing with a broad base, and standing with arms overhead were given focusing on the body sensations and thoughts	2–3 min
	Lying Calmly	Lying in a supine posture with eyes closed focusing on breathing and the positive thoughts	3–4 min
	Mindfully walking	Walking in a calm area by keeping the awareness of the physical sensations and engaging the senses by looking and feeling the different senses	3–4 min
Progressive muscle relaxation	Systematic muscle tensing and relaxation	Starting from toes to head for tensing each muscle group for 5–10 secs and sequentially promoting the same	3–4 min
	Guided imagery	Envision of mind to a calm scene and scenario and engage the senses to bring life into it	3–4 min
Vocal toning exercises	Tongue trills	Touching the tongue on top of front teeth and making the sound of "I"	1–2 min
	Humming sounds	Sitting in a relaxed position and making humming sounds by keeping mouth closed	(10–12 breaths)1–2 min
	Vowel sounds	In relaxed sitting, during exhalation making a long vowel sound	(10–12 breaths)1–2 min
Self-soothing touch exercises	Foot massage	Massaging your foot while paying attention to the sensations and feeling of touch	1–2 min
	Havening touch	Sitting in a calm place, keeping a positive mind away from distractions and touching your arms, face, and palms	1–2 min
	Belly breathing	Place one hand on your abdomen, just below the ribcage. Take slow, deep breaths, allowing your belly to rise and fall with each inhalation and exhalation	1–2 min

#### Statistical analysis

In this study, data analysis was conducted using SPSS version 23.0 (IBM Corp, Armonk, NY). Descriptive statistics, such as the mean and standard deviation (SD), were employed to present quantitative variables. The normality of data was assessed using the Shapiro‒Wilk test. An independent t test was applied to assess whether the means of the two groups were significantly different from each other, given the variation within each group. Cohen's d was employed to estimate effect size, with a confidence interval of 95% and a significance level of *p* < 0.05. Based on Cohen's d as a measure of effect size, the differences were classified as small (d < 0.2), moderate (0.2 < d < 0.5) and large (d ≥ 0.5). Additionally, a 2 (group) × 2 (time) repeated-measures ANOVA was utilized to examine the simple effects of time and group as well as the time × group interaction effect on outcome variables. Subsequently, post hoc pairwise comparisons were conducted to further investigate significant findings.

### Ethical approval and consent to participate

The study protocol conformed to the ethical guidelines of the 1975 Declaration of Helsinki and was reviewed and approved by the ethics subcommittee of King Saud University, Riyadh, Saudi Arabia, under file ID RRC-2019-14, dated 22-04-2019. In addition, the Institutional Ethical Committee at Manav Rachna International Institute of Research & Studies approved the study protocol under reference number EC/2023-24/033, since the participants included patients visiting the OPD. Each participant was asked to submit a signed informed consent form as proof of consent to participate in the study. To ensure transparency and accountability, the study protocol was registered in the clinical trial registry at https://www.ctri.nic.in/ with the identifier CTRI/2023/08/056055.

## Results

Table 3 presents the results of an independent t test to assess whether the mean values of the two groups (Group 1 and Group 2) were significantly different from each other for various outcome variables, specifically WOMAC (Western Ontario and McMaster Universities Osteoarthritis Index) and WHOQOL (World Health Organization Quality of Life) scores. On a comparison of the mean values of WOMAC subscales, although there was no significant difference in joint pain (t = − 1.06, *p* = 0.30), joint stiffness (t = − 1.08, *p* = 0.29) or functional limitations (t = − 1.38, *p* = 0.17) before the intervention, Group 2 demonstrated significantly greater reductions in joint pain (t = 11.55, *p* < 0.001), joint stiffness (t = 2.5, *p* = 0.02) and functional limitations (t = 5.17, *p* < 0.001) than Group 1 after the intervention. The effect size suggests a large difference, with Group 2 experiencing more substantial decreases in joint pain (d = 3.27), joint stiffness (d = 0.71) and functional limitations (d = 1.46). There was no significant difference in the mean WOMAC Index scores between Group 1 (68.60 ± 5.07) and Group 2 (71.32 ± 5.93) before the intervention (t = − 2.00, *p* = 0.05). However, after the intervention, Group 2 (30.60 ± 5.55) showed a significantly greater improvement in WOMAC scores than Group 1 (43.72 ± 5.12) (t = 8.69, *p* < 0.001). The effect size (Cohen's d) was 2.46, indicating a large difference between the groups. This suggests that Group 2 exhibited outcomes that were more favorable and were more effective in reducing the impact of the condition being assessed.

**Table 3:** Independent t test applied to compare the mean WOMAC and WHOQOL scores between Group 1 and Group 2.

Outcome variables		Group 1 Mean ± SD	Group 2 Mean ± SD	t	*p*	Cohen's d	95% CI	
							Lower	Upper
WOMAC								
Joint pain	Pre	15.40 ± 2.00	16.00 ± 2.02	− 1.06	0.30	− 0.30	− 0.85	0.26
		10.60 ± 1.73	5.72 ± 1.21	11.55	**< 0.001**	3.27	2.40	4.12
Stiffness	Pre	5.76 ± 1.36	6.24 ± 1.76	− 1.08	0.29	− 0.3	− 0.86	0.25
		2.88 ± 1.05	2.24 ± 0.72	2.5	**0.02**	0.71	0.13	1.28
Functional limitation	Pre	47.04 ± 4.75	49.08 ± 5.63	− 1.38	0.17	− 0.39	− 0.95	0.17
		30.24 ± 4.74	22.64 ± 5.62	5.17	**< 0.001**	1.46	0.83	2.08
WOMAC index	Pre	68.60 ± 5.07	71.32 ± 5.93	− 2.00	0.05	− 0.57	− 1.13	0.00
		43.72 ± 5.12	30.60 ± 5.55	8.69	**< 0.001**	2.46	1.71	3.19
WHOQoL								
Overall QoL	Pre	2.8 ± 0.87	2.4 ± 0.71	1.79	0.08	0.51	− 0.06	1.07
	Post	3.56 ± 1.08	3.64 ± 0.86	− 0.29	0.77	− 0.08	− 0.64	0.47
General health	Pre	2.6 ± 0.82	2.52 ± 0.82	0.35	0.73	0.1	− 0.46	0.65
	Post	3.2 ± 1.08	3.72 ± 0.94	− 1.82	0.08	− 0.51	− 1.08	0.05
Physical domain	Pre	11.92 ± 1.19	12.52 ± 1.56	− 1.53	0.13	− 0.43	− 0.99	0.13
	Post	16.08 ± 1.44	15.68 ± 1.65	0.91	0.37	0.26	− 0.3	0.81
Psychological domain	Pre	14.36 ± 1.7	14.92 ± 1.08	− 1.39	0.17	− 0.39	− 0.95	0.17
	Post	15.24 ± 1.69	17.84 ± 0.94	− 6.72	**< 0.001**	− 1.9	− 2.56	− 1.22
Social domain	Pre	14.52 ± 0.96	14.48 ± 1.39	0.12	0.91	0.03	− 0.52	0.59
	Post	15.04 ± 0.98	17.48 ± 0.96	− 8.89	**< 0.001**	− 2.51	− 3.25	− 1.76
Environmental domain	Pre	13.92 ± 1.75	13.28 ± 1.62	1.34	0.19	0.38	− 0.18	0.94
	Post	15.92 ± 1.26	16.48 ± 1.19	− 1.62	0.11	− 0.46	− 1.02	0.11

*WOMAC* Western Ontario and McMaster Universities Osteoarthritis Index, *WHOQoL BREF* WHO Quality of Life BREF.

Significant values are in [bold].

Furthermore, independent t tests were conducted to compare the mean values of the WHOQOL variable; there were no significant differences in the ratings of overall quality of life and general health between the pre- and post-intervention timepoints in Group 1 and Group 2. In a comparison of the physical and environmental subdomains of the WHOQOL between Group 1 and Group 2, the difference was not significant for either pre- or post-intervention assessments. In a comparison of the psychological subdomain of the WHOQOL between Group 1 and Group 2, for the pre-intervention assessment, the difference in the mean scores was not statistically significant (t = − 1.39, *p* = 0.17), and the effect size was small. However, for the post-intervention assessment, there was a significant difference in the mean scores for the psychological domain between Group 1 and Group 2 (t = − 6.72, *p* < 0.001), with a large effect size (Cohen's d = − 1.9). Additionally, in the social domain of the WHOQOL, the two groups presented similar results in the pre-intervention assessment, with no significant difference in the mean scores (t = 0.12, *p* = 0.91) between Group 1 and Group 2. However, for the post-intervention assessment, there was a significant difference in the mean scores for the social domain between Group 1 and Group 2 (t = − 8.89, *p* < 0.001), with a large effect size (Cohen's d = − 2.51), indicating a substantial difference.

The t test results indicate that there were no significant differences in resting HR between Group 1 and Group 2 at either the pre-intervention or post-intervention assessment (Table 4). There were significant differences in the RR interval between Group 1 and Group 2 at both the pre- (t = 3.3, *p* < 0.001) and post-intervention (t = − 2.65, *p* = 0.01) assessments. Furthermore, the independent t test results indicated no significant differences between Group 1 and Group 2 in terms of SDNN or RMSSD measures at either timepoint.

**Table 4:** Independent t test applied to compare the mean values of HRV (time and frequency domains) between Group 1 and Group 2.

HRV		Group 1 Mean ± SD	Group 2 Mean ± SD	t	*p*	Cohen's d	95% CI	
							Lower	Upper
Time domain								
Resting HR (beat/min)	Pre	82.48 ± 6.46	81.64 ± 4.88	0.52	0.61	0.15	− 0.41	0.7
	Post	80.48 ± 6.1	79.6 ± 6.06	0.51	0.61	0.14	− 0.41	0.7
Mean RR (ms)	Pre	711.55 ± 62.96	662.32 ± 39.87	3.3	**< 0.001**	0.93	0.34	1.51
	Post	742.05 ± 62.58	786.93 ± 57.21	− 2.65	**0.01**	− 0.75	− 1.32	− 0.17
SDRR (ms)	Pre	112.91 ± 9.8	113.37 ± 10.6	− 0.16	0.87	− 0.05	− 0.6	0.51
	Post	116.91 ± 10.85	119.82 ± 11.37	− 0.92	0.36	− 0.26	− 0.82	0.3
RMSSD	Pre	21.22 ± 5.97	19.97 ± 4.37	0.85	0.40	0.24	− 0.32	0.8
	Post	24.39 ± 6.71	24.26 ± 6.12	0.07	0.94	0.02	− 0.53	0.57
Frequency domain								
LF (ms^2^)	Pre	533.55 ± 63.53	550.12 ± 95.17	− 0.72	0.47	− 0.2	− 0.76	0.35
	Post	543.55 ± 63.53	521.34 ± 48.17	1.39	0.17	0.39	− 0.17	0.95
HF (ms^2^)	Pre	295.23 ± 72.19	286.68 ± 90.47	0.37	0.71	0.1	− 0.45	0.66
	Post	334.58 ± 50.99	392.16 ± 48.88	− 4.08	**< 0.001**	− 1.15	− 1.75	− 0.55
TP (ms^2^)	Pre	1360.5 ± 209.47	1355.17 ± 327.33	0.07	0.95	0.02	− 0.54	0.57
	Post	1474.4 ± 246.23	1449.41 ± 201.75	0.39	0.70	0.11	− 0.44	0.67
LF/HF	Pre	1.89 ± 0.42	2.04 ± 0.52	− 1.1	0.28	− 0.31	− 0.87	0.25
	Post	1.66 ± 0.37	1.34 ± 0.18	3.87	**< 0.001**	1.09	0.49	1.69
nuLF	Pre	39.55 ± 3.64	41.19 ± 4.3	− 1.46	0.15	− 0.41	− 0.97	0.15
	Post	37.55 ± 5.98	36.37 ± 4.16	0.81	0.42	0.23	− 0.33	0.78
nuHF	Pre	21.56 ± 3.64	21.09 ± 4.03	0.44	0.66	0.12	− 0.43	0.68
	Post	23.04 ± 3.81	27.41 ± 4.17	− 3.87	**< 0.001**	− 1.1	− 1.69	− 0.49

*mRR* mean RR interval, *SDNN* standard deviation of normal-to-normal intervals, *RMSSD* root mean square of successive differences, *LF* low-frequency spectral power, *HF* high-frequency spectral power, *LF/HF* ratio of low-frequency power to high-frequency power, *TP* total power, *nu* normalized units.

Significant values are in [bold].

Independent t tests showed that there were no significant differences between Group 1 and Group 2 in LF, TP, nuLF, or nuHF at either the pretreatment or posttreatment timepoint. There was no significant difference in the pre-intervention comparison of HF between Group 1 (295.23 ± 72.19) and Group 2 (286.68 ± 90.47) (t = 0.37, *p* = 0.71, d = 0.1). However, Group 2 showed a significant increase in HF compared to Group 1 (t = − 4.08, *p* < 0.001, d = − 1.15) in the post-intervention assessment.

Before the intervention, there was no significant difference in the LF/HF ratio between Group 1 (1.89 ± 0.42) and Group 2 (2.04 ± 0.52) (t = − 1.1, *p* = 0.28, d = − 0.31). However, after the intervention, there was a significant difference in the LF/HF ratio between the groups (t = 3.87, *p* < 0.001, d = 1.09). Before the intervention, there were no significant differences in nuHF (Group 1: 21.56 ± 3.64, Group 2: 21.09 ± 4.03) between the groups (nuHF: t = 0.44, *p* = 0.66, d = 0.12). This suggests that the two groups initially had similar normalized HF values. After the intervention, there was a significant difference in nuHF (t = − 3.87, *p* < 0.001, d = − 1.1) between the groups.

Table 5 presents the results of repeated-measures ANOVA comparing outcome measures between Group 1 and Group 2, with time (pre- or post-intervention measurement) as the within-subjects factor and the outcome measure as the between-subjects factor.

**Table 5:** A repeated-measures ANOVA to compare outcome measures between two groups (1 and 2), with time (pre- or post-intervention measurement) as the within-subjects factor and the outcome measure as the between-subjects factor.

Outcome variables	Group 1		Group 2		Time (T) effect ηp2 (*p*-value)	Group (G) effect ηp2 (*p*-value)	T x G interaction ηp2 (*p*-value)
	Pre	Post	Pre	Post			
ROM							
ROM	100.16 ± 9.95	118.24 ± 9.18^a^	101 ± 7.36	123 ± 7.64^b^	**0.87 (*p* < 0.001 )**	0.03 (0.20)	0.06 (0.08)
WOMAC							
Joint pain	15.40 ± 2.00	10.60 ± 1.73^a^	16.00 ± 2.02	5.72 ± 1.21^b^	**0.96 (*p* < 0.001)**	**0.32 (*p* < 0.001)**	**0.76 (*p* < 0.001)**
Stiffness	5.76 ± 1.36	2.88 ± 1.05^a^	6.24 ± 1.76	2.24 ± 0.72^b^	**0.91 (*p* < 0.001)**	0.0001 (0.81)	**0.22 (*p* < 0.001)**
Functional limitation	47.04 ± 4.75	30.24 ± 4.74^a^	49.08 ± 5.63	22.64 ± 5.62^b^	**0.94 (*p* < 0.001)**	**0.09 (0.03)**	**0.45 (*p* < 0.001)**
WOMAC Index	68.6 ± 5.13	37.96 ± 4.89^a^	71.68 ± 6.09	31.08 ± 5.54^b^	**0.97 (*p* < 0.001)**	**0.24 (*p* < 0.001)**	**0.67 (*p* < 0.001)**
WHOQoL							
Overall QoL	2.8 ± 0.87	3.56 ± 1.08^a^	2.4 ± 0.71	3.64 ± 0.86^b^	**0.85 (*p* < 0.001)**	0.0001 (0.51)	**0.24 (*p* < 0.001)**
General health	2.6 ± 0.82	3.2 ± 1.08^a^	2.52 ± 0.82	3.72 ± 0.94^b^	**0.61 (*p* < 0.001)**	0.02 (0.36)	**0.15 (*p* < 0.001)**
Physical domain	11.92 ± 1.19	16.08 ± 1.44^a^	12.52 ± 1.56	15.68 ± 1.65^b^	**0.79 (*p* < 0.001)**	0.001 (0.75)	0.06 (0.08)
Psychological domain	14.36 ± 1.7	15.24 ± 1.69	14.92 ± 1.08	17.84 ± 0.94^b^	**0.77 (*p* < 0.001)**	**0.28 (*p* < 0.001)**	**0.49 (*p* < 0.001)**
Social domain	14.52 ± 0.96	15.04 ± 0.98	14.48 ± 1.39	17.48 ± 0.96^b^	**0.69 (*p* < 0.001)**	**0.31 (*p* < 0.001)**	0.52 (0.08)
Environmental domain	13.92 ± 1.75	15.92 ± 1.26^a^	13.28 ± 1.62	16.48 ± 1.19^b^	**0.72 (*p* < 0.001)**	0.0001 (0.91)	**0.12 (0.01)**
HRV time domain							
Resting HR (beats/min)	82.48 ± 6.46	80.48 ± 6.1	81.64 ± 4.88	79.6 ± 6.06	**0.08 (0.04)**	0.001 (0.53)	0.001 (0.98)
Mean RR (ms)	711.55 ± 62.96	742.05 ± 62.58	662.32 ± 39.87	786.93 ± 57.21^b^	**0.76 (*p* < 0.001)**	0.0001 (0.88)	**0.53 (*p* < 0.001)**
SDNN (ms)	112.91 ± 9.8	116.91 ± 10.85	113.37 ± 10.6	119.82 ± 11.37^b^	**0.13 (0.01)**	0.01 (0.46)	0.001 (0.54)
RMSSD	21.22 ± 5.97	24.39 ± 6.71	19.97 ± 4.37	24.26 ± 6.12^b^	**0.64 (*p* < 0.01)**	0.0001 (0.67)	0.04 (0.17)
HRV frequency domain							
LF (ms^2^)	533.55 ± 63.53	543.55 ± 63.53	550.12 ± 95.17	521.34 ± 48.17	0.02 (0.32)	0.0001 (0.87)	**0.08 (0.04)**
HF (ms^2^)	295.23 ± 72.19	334.58 ± 50.99^a^	286.68 ± 90.47	392.16 ± 48.88^b^	0.02 (0.32)	0.0001 (0.87)	**0.08 (0.04)**
TP (ms^2^)	1360.5 ± 209.47	1474.4 ± 246.23	1355.17 ± 327.33	1449.41 ± 201.75	**0.14 (*p* < 0.001)**	0.00001 (0.80)	0.0001 (0.79)
LF/HF	1.89 ± 0.42	1.66 ± 0.37	2.04 ± 0.52	1.34 ± 0.18^b^	**0.43 (*p* < 0.001)**	0.02 (0.29)	**0.16 (0.0001)**
nuLF	39.55 ± 3.64	37.55 ± 5.98	41.19 ± 4.3	36.37 ± 4.16^b^	**0.25 (*p* < 0.001)**	0.0001 (0.81)	0.05 (0.11)
nuHF	21.56 ± 3.64	23.04 ± 3.81	21.09 ± 4.03	27.41 ± 4.17^b^	**0.43 (*p* < 0.001)**	**0.09 (0.04)**	**0.23 (*p* < 0.001)**

‘a’ shows the significant difference between pre to post-measurement in Group 1 while ‘b’ shows difference in Group 2. *mRR* mean RR interval, *SDNN* standard deviation of normal-to-normal intervals, *RMSSD* root mean square of successive differences, *LF* low-frequency spectral power, *HF* high-frequency spectral power, *LF/HF* ratio of low-frequency power to high-frequency power, *TP* total power, *nu* normalized units.

Significant values are in [bold].

Both groups showed significant improvements in range of motion (ROM) after the intervention, with the mean difference in ROM being greater in Group 2. There was a significant effect of time, suggesting an overall improvement in ROM. Both Group 1 and Group 2 experienced significant improvements in joint pain, stiffness, functional limitations, and the WOMAC index. Group 2 exhibited larger mean differences and more substantial improvements in all assessed outcome measures than Group 1.

Both groups showed significant improvements in overall quality of life; general health; and the physical, psychological, social, and environmental domains. The time effects were significant, indicating an overall improvement in quality of life across all domains. Group effects were significant for the psychological domain and the social domain.

For resting HR (beats/min), there was a significant time effect between the pre- and post-intervention timepoints; however, the group effect (Group 1 vs. 2) was not significant. Regarding the time-domain HRV measures of Mean RR (ms), SDNN (ms), and RMSSD, both Group 1 and Group 2 showed significant improvements (a time effect) after the intervention. The interaction effects were significant for Mean RR, indicating that the change in Mean RR differed between the two groups. Comparing the mean differences, it appears that Group 2 had larger improvements in Mean RR, SDNN, and RMSSD than Group 1. Therefore, Group 2 generally showed changes that were more favorable in the HRV time domain variables, indicating a potential improvement in cardiovascular health. In the HRV frequency-domain analysis, there were no significant time or group effects for LF or HF. There was a significant time effect of TP but no group effect or time × group interaction. There was a significant time effect as well as a significant time × group interaction for the LF/HF ratio. There was a significant time effect for nuLF but no significant group or interaction effect. On the other hand, nuHF showed a significant time effect, group effect and time × group interaction.

## Discussion

This study aimed to compare the effects of knee strengthening exercises (Group 1) and exercises based on polyvagal theory combined with knee strengthening exercises (Group 2) in females with grade II knee OA. The study assessed various outcome variables, including WOMAC scores (joint pain, joint stiffness, functional limitations, and the overall index) and WHOQOL scores measuring QoL; general health; and the physical, psychological, social, and environmental domains. Additionally, autonomic function was measured by heart rate variability (HRV) measures, which were analyzed in the time and frequency domains.

Polyvagal theory, proposed by Porges, suggests that the vagus nerve plays a crucial role in regulating the autonomic nervous system and influencing emotional states, stress responses, and social engagement. Engaging in activities that stimulate the vagus nerve, such as the exercises described, may promote relaxation, reduce anxiety, and enhance emotional regulation. These outcomes could ultimately contribute to a reduction in perceived pain and an increase in quality of life in patients with knee osteoarthritis^9^. Vagus nerve stimulation has multifaceted mechanisms that could explain its potential benefits for individuals with knee osteoarthritis. It activates the parasympathetic nervous system, increasing vagal tone while reducing sympathetic activity, which might lead to pain reduction and enhanced well-being^9^. Moreover, vagal stimulation exhibits anti-inflammatory effects, regulating immune responses and potentially alleviating arthritic joint inflammation, thus contributing to pain relief^22^. Additionally, by influencing central pain mechanisms and neural pathways, VNS can modulate pain perception and processing, thereby potentially enhancing pain relief^22^. Finally, vagal stimulation's impact on neuroplasticity could lead to lasting improvements in pain and quality of life for those with knee osteoarthritis^5^.

The WOMAC assessment for knee OA is a sensitive tool, well suited for detecting changes in symptoms and function over time or in response to interventions. This evaluation captures the patient's perspective, facilitating a comprehensive assessment of the impact of knee OA on daily life and functioning^16^. In the present study, both groups displayed effects as measured by WOMAC subscales; however, the group that performed VNS exercises demonstrated notable improvements in pain, stiffness and functional limitations. Notably, the results indicated that Group 2 exhibited significantly greater reductions in joint pain, joint stiffness, and functional limitations than Group 1 after 6 weeks. Group 2 also exhibited significant improvements in the WOMAC index scores, reflecting favorable overall outcomes. In terms of quality of life, Group 2 demonstrated marked enhancements in the psychological and social domains compared to Group 1. However, there were no significant differences observed in the physical domain or the environmental domain.

Muscle and skeletal problems constitute over a quarter of nonlethal health losses, with pain being the most significant contributor^23^. Chronic pain related to OA not only restricts social functioning but also heightens the risk of psychological issues^24^, depression^25^ and diminished work capabilities^26^. The finding of this study underscores the favorable response observed with polyvagal exercises, which predominantly focus on relaxation. As a nonpharmacological intervention, relaxation is increasingly employed to alleviate pain and enhance pain management^27^. This finding aligns with Turk and Winter’s research, illustrating that achieving a state of physical and mental relaxation influences psychological and bodily well-being, fostering calmness and contributing to the reduction of pain^28^. Further reinforcement is derived from a study by Onivea-Zefra et al., who demonstrate the potential of guided imagery relaxation in reducing pain levels among fibromyalgia patients. Through the utilization of visualizations and mental imagery techniques, this approach promotes relaxation and helps in alleviating pain perception^29^.

Another study by Morone and Greco reinforces the findings that guided imagery and relaxation offer clinical pain management benefits for individuals with knee OA^30^. The reduction in pain, functional limitations and stiffness could also be attributed to the effects of strengthening exercises. Strength training has been linked to potential disease-modifying impacts in OA. Regular exercise can promote joint health, stimulate cartilage metabolism, and improve muscle strength and joint stability^31^.

In individuals with osteoarthritis, pain is the most prevalent complaint and a primary contributor to reduced health-related quality of life (HRQoL). In this study, both groups demonstrated significant improvements across various domains of quality of life, including overall quality; general health; and physical, psychological, social, and environmental aspects. Notably, Group 2 exhibited more substantial enhancements in overall quality of life, general health, psychological, social, and environmental domains than Group A, which underwent vagal nerve stimulation-based exercise. The improvement in the physical domain is attributed to the 6-week exercise regimen, a sentiment that aligns with Smith et al.’s findings, emphasizing the efficacy of a scalable exercise program in enhancing the well-being and function of hip and knee OA patients^32^. Various techniques, including deep breathing, meditation, relaxation exercises, and specific physical activities, can stimulate the vagus nerve and enhance its functionality. These exercises may facilitate relaxation, stress reduction, and overall well-being. Goff et al.’s findings agree with ours, suggesting that patient education positively impacts conservative treatment and can be productively used in concert with it^33^. Our results are corroborated by Ebenezer et al.’s study, which revealed that patients treated with yoga experienced substantial improvements in QoL, paralleling the effects of therapeutic exercise on OA patients. The shared impact of yoga on emotional stability and quality of life, a factor pivotal in pain reduction and enhanced quality of life, is a significant highlight of their research^34^.

Vagal stimulation, in line with the polyvagal theory, is also emphasized by Wang et al., who suggest that practices such as yoga—a form of physical and relaxation exercise—have a systemic effect. By stimulating parasympathetic pathways, yoga reduces perceived pain and improves QoL^35^. Regarding heart rate variability (HRV), Group 2 exhibited a notable increase in high-frequency power (HF) and a significant decrease in the LF/HF ratio, indicating enhanced autonomic regulation. These outcomes imply that the combination of polyvagal exercises and knee-strengthening training yields a favorable impact on HRV, reflecting improved cardiovascular health. Interestingly, a positive correlation exists between disability and cardiovascular health in patients with knee OA. This is underscored by Tsubio et al.’s discovery of a connection between degenerative diseases and causes of death in Japan, revealing that individuals with knee OA are at an elevated risk of heart attacks leading to mortality^36^. This underscores the imperative for enhanced autonomic regulation in knee OA patients.

The observed changes in HRV parameters can be attributed to the stimulation of the vagal nerve. This emphasizes the contention of the polyvagal theory that the physiological state is not merely a correlate but a fundamental component of emotions and moods. Within this theoretical framework, the autonomic state functions as an intermediary variable that influences our perception and evaluation of environmental stimuli. The reflexive assessment of cues as neutral, positive or threatening can vary based on an individual's physiological state. Functionally, changes in an individual’s state can lead to a shift in the accessibility of different brain structures, facilitating social communication or eliciting defensive responses such as fight/flight or shutdown^37^. Goldbeck et al. also emphasized the importance of yoga-based exercises in enhancing HRV parameters, consequently improving parasympathetic activity^38^.

The study had a few limitations that should be noted. It had a small sample size, exclusively female participants, and a lack of long-term follow-up. While generalization to the wider population should be approached with caution, the study's findings still contribute to a broader understanding of the potential benefits of the interventions within this specific subset of patients. The decision to include female participants only was driven by the fact that knee osteoarthritis is more commonly observed in females than in males. Evaluating the sustainability and durability of the benefits conferred by polyvagal exercises in the long term could provide a more comprehensive understanding of their effects and guide clinical recommendations. Furthermore, there was no control group, and blinding procedures were not implemented. The inability to implement blinding in this study is attributable to the nature of the interventions employed, which made it a practical challenge to blind participants and researchers to the group assignments, as the participants themselves needed to be aware of the specific exercises they were performing. The study relied on self-reported measures and did not use objective measures. A total reliance on self-reported measures, without the use of objective assessments, introduces the potential for measurement bias due to participants' subjective perceptions and recall biases. Integrating objective measures such as biomechanical assessments or biomarkers would offer a more comprehensive understanding of the interventions' effects. Finally, the dropout rate was 10%, which may have affected the representativeness of the final sample and introduced potential bias.

## Conclusions

The study highlights the potential benefits of combining strengthening exercises with exercises based on polyvagal theory in the management of grade II knee OA. Strengthening exercises can improve joint stability and reduce stress on the joint, while exercises based on polyvagal therapy may help regulate autonomic function, reduce pain perception, and promote relaxation and emotional well-being. The combination of these interventions may lead to superior overall outcomes and improved quality of life for individuals with knee osteoarthritis. These results support the potential effectiveness of incorporating polyvagal exercise as an adjunctive intervention for osteoarthritis management.

### Future scope

The study opens up several potential avenues for future research. First, conducting a larger-scale study with a more diverse participant pool, including both genders, would help establish the generalizability of the findings. Long-term follow-up assessments could be incorporated to examine the sustainability and durability of the effects observed. Additionally, incorporating a control group and implementing blinding procedures would enhance the study's internal validity. Objective measures, such as physiological recordings, could be used in conjunction with self-report measures to provide a more comprehensive understanding of the outcomes. Exploring different stimulation parameters and investigating the mechanisms underlying vagal nerve stimulation could further elucidate its therapeutic potential. Overall, future research should build upon these limitations to strengthen the evidentiary base and better inform clinical applications of VNS.

## Data Availability

All data generated or analyzed during this study are presented in the manuscript. Please contact the corresponding author for access to the data from this study.
